# Cyclic Hypoxia Exposure Accelerates the Progression of Amoebic Gill Disease

**DOI:** 10.3390/pathogens9080597

**Published:** 2020-07-22

**Authors:** Tina Oldham, Tim Dempster, Philip Crosbie, Mark Adams, Barbara Nowak

**Affiliations:** 1 Institute of Marine Research, 5984 Matre, Norway; 2 Aquatic Animal Health Group, Institute for Marine and Antarctic Studies, University of Tasmania, Launceston, TAS 7248, Australia; philip.crosbie@utas.edu.au (P.C.); mark.adams@utas.edu.au (M.A.); b.nowak@utas.edu.au (B.N.); 3 Sustainable Aquaculture Laboratory–Temperate and Tropical (SALTT), School of BioSciences, University of Melbourne, Parkville, VIC 3010, Australia; dempster@unimelb.edu.au

**Keywords:** *Salmo salar*, Atlantic salmon, aquaculture, dissolved oxygen, *Paramoeba/Neoparamoeba perurans*, stress

## Abstract

Amoebic gill disease (AGD), caused by the amoeba *Neoparamoeba perurans*, has led to considerable economic losses in every major Atlantic salmon producing country, and is increasing in frequency. The most serious infections occur during summer and autumn, when temperatures are high and poor dissolved oxygen (DO) conditions are most common. Here, we tested if exposure to cyclic hypoxia at DO saturations of 40–60% altered the course of infection with *N. perurans* compared to normoxic controls maintained at ≥90% DO saturation. Although hypoxia exposure did not increase initial susceptibility to *N. perurans*, it accelerated progression of the disease. By 7 days post-inoculation, amoeba counts estimated from qPCR analysis were 1.7 times higher in the hypoxic treatment than in normoxic controls, and cumulative mortalities were twice as high (16 ± 4% and 8 ± 2%), respectively. At 10 days post-inoculation, however, there were no differences between amoeba counts in the hypoxic and normoxic treatments, nor in the percentage of filaments with AGD lesions (control = 74 ± 2.8%, hypoxic = 69 ± 3.3%), or number of lamellae per lesion (control = 30 ± 0.9%, hypoxic = 27.9 ± 0.9%) as determined by histological examination. Cumulative mortalities at the termination of the experiment were similarly high in both treatments (hypoxic = 60 ± 2%, normoxic = 53 ± 11%). These results reveal that exposure to cyclic hypoxia in a diel pattern, equivalent to what salmon are exposed to in marine aquaculture cages, accelerated the progression of AGD in post-smolts.

## 1. Introduction

Metabolically limiting dissolved oxygen (DO) conditions, termed hypoxic, occur in Atlantic salmon (*Salmo salar*) cages around the world, and are predicted to increase in frequency and severity as the global climate warms [1,2,3,4]. In the wild, limited evidence suggests that salmon avoid areas with low DO [5]. In marine cages however, during the seawater grow-out phase of production, the response of salmon to low DO is variable. When faced with severely hypoxic conditions of <30% DO saturation, 40% of the salmon tagged in two Tasmanian cages died, while the surviving individuals prioritized avoidance of the most hypoxic areas over other environmental and social factors [4]. In several other marine cage studies, salmon remained in moderately hypoxic waters at levels known to reduce feed intake, growth, and immune competence, despite other areas of the cage having higher DO [3,6,7].

Given the frequency of occurrence and regular exposure of salmon to hypoxia, our understanding of how such conditions influence susceptibility to disease is inadequate [8]. All activity is limited by the availability of energetic resources. In salmon, the primary means of energy generation is aerobic metabolism, a process which requires DO [9]. When the amount of environmental DO available is insufficient to meet metabolic demand, any activity not essential to immediate survival is minimized [10]. For salmon in moderately hypoxic conditions (45–55% DO saturation at 16 °C), the aerobic scope for activity of post-smolts was only 38% of what it was in normoxia (85–95% DO saturation), effectively halving the energetic resources available [11]. More extreme hypoxia would decrease aerobic scope even further.

As aerobic scope declines, energetic prioritization becomes necessary. One of the primary responses to stress in salmon is increased circulating cortisol concentration [12,13]. In resting conditions cortisol maintains homeostasis; in stressful conditions, however, cortisol mobilizes energetic resources by enhancing gluconeogenesis while suppressing reproductive functions and immune response [14]. In post-smolt Atlantic salmon, exposure to moderate, intermittent hypoxia leads to increased plasma cortisol concentrations and significantly reduced leucocyte function and thus putatively weakened innate immunity [12,15]. Exposure to chronic hypoxia causes reduced and/or delayed expression of several immune related genes, even after cortisol levels return to normal [13]. 

Beyond the basic physiological impacts, evidence suggests that the stress and reduced immune competence caused by hypoxia exposure result in increased susceptibility to diseases in fish. For example, while no Nile Tilapia (*Oreochromis niloticus*) maintained in normoxic conditions died as a result of injection with *Streptococcus agalactiae*, 80% mortality occurred in fish exposed to sub-lethal hypoxia for 24 h prior to infection [16]. Similar results were observed in Channel Catfish (*Ictalurus punctatus*)- groups exposed to 2 h of sub-lethal hypoxia immediately prior to infection challenge with *Edwardsiella ictaluri* experienced significantly higher cumulative mortality (36%) than those maintained in normoxic conditions (12%) [17].

The influence of hypoxia on disease susceptibility in Atlantic salmon, however, remains unclear. Though cortisol is known to affect immune response in salmon [18], in a trial which exposed post-smolts to constant, mild hypoxia (60–65% saturation at 12 °C) after exposure to salmonid alphavirus, no differences in disease progression or prognosis were observed over a 70 day period [19]. Similarly, when salmon were exposed to 4 h of hypoxia three times throughout 10 weeks after infection with *Piscine orthoreovirus* (PRV), no difference in infection level or histopathology were observed between DO treatment groups at any time-point. However, during peak pathologythe hypoxia tolerance of infected fish which had been previously exposed to hypoxia was the same as un-infected controls, and significantly better than the infected fish maintained in normoxic conditions [20]. No studies have investigated the influence of periodic, moderately hypoxic conditions, such as those commonly observed in marine cages, on disease progression in salmon. 

Many pathogens threaten salmon during the seawater production phase, but the cosmopolitan distribution and lethality of amoebic gill disease (AGD) make it one of the biggest threats to salmon welfare globally [21]. Caused by a parasitic amoeba, *Neoparamoeba perurans*, AGD is characterized by epithelial hyperplasia and lamellar fusion of gills resulting in compromised gill function and reduced aerobic scope [22,23,24]. Given the focused pathological impact of *N. perurans* on gills and relevance to the global industry, understanding the role of hypoxia in AGD susceptibility and progression can help producers to adopt appropriate management strategies and optimize treatment regimes. Here, we examine if cyclic exposure to moderate hypoxia, similar to conditions documented in marine aquaculture cages, alters AGD acquisition, progression, and prognosis in post-smolt Atlantic salmon.

## 2. Results

### 2.1. Amoeba Acquisition and Proliferation

**Figure 1:**
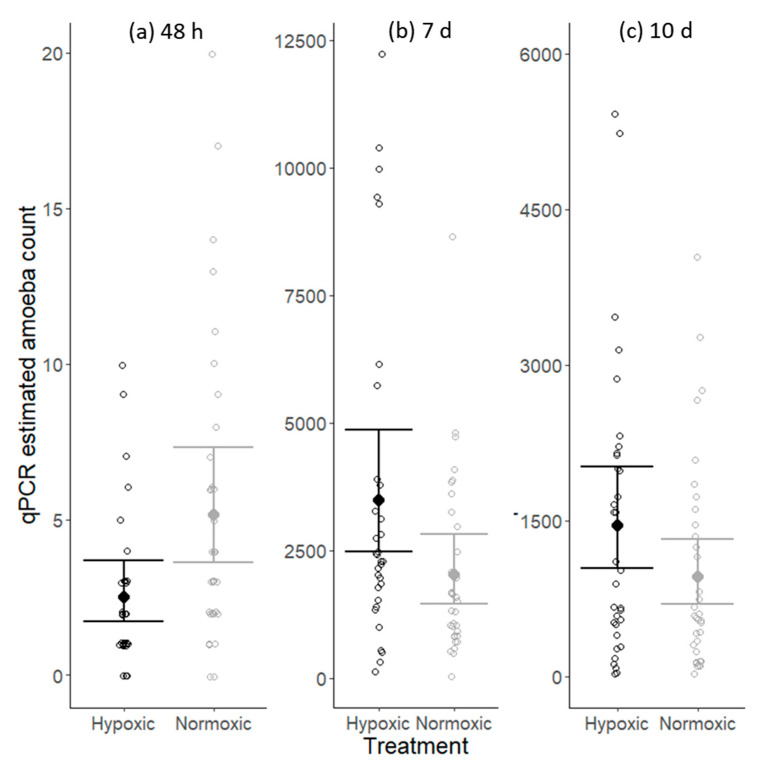
Influence of dissolved oxygen (DO) treatment and time since inoculation on qPCR estimated amoeba counts at 48 h (**a**), 7 d (**b**) and 10 d (**c**) post-inoculationOpen points are estimated amoeba counts for each fish tested, while solid points and error bars display the fitted generalized linear mixed model (GLMM) predicted means with 95% confidence intervals. The hypoxic treatment is black and the normoxic treatment is grey.

*N. perurans* were detected on the gills of individuals in both the normoxic and hypoxic treatments at all three sample collection periods. At the first sampling, 48 h post-infection, amoeba numbers were low in both treatments, with estimates ranging from 0 to a maximum of 20 cells. By the 2nd sampling, 7 days post-inoculation, amoeba numbers had increased considerably and ranged from 123 to 12,231 in the hypoxic treatment, and 36 to 8657 in normoxic. The median in the hypoxic treatment was 2413 amoebae, while in the normoxic treatment it was 1449. Due to using a different sampling technique, whole arch processing rather than mucous swabs, the amoeba numbers from the 3rd sampling are not directly comparable to the previous two sampling points. However, at this sampling, amoeba numbers were still high in both treatments and not significantly different (Figure 1).

The number of *N. perurans* cells detected on gills was significantly influenced by DO treatment and time since inoculation (Table 1), with a conditional r-squared value of 0.90. Two days after inoculation more *N. perurans* cells were detected on fish in the normoxic DO treatment than in hypoxic (Figure 1a). By 7 days post-inoculation, amoeba numbers had increased substantially and were 1.7 times higher in the hypoxic DO treatment than in the normoxic group (Figure 1b). At the final sampling, 10 days post-inoculation, there was no significant difference between treatment groups in the number of *N. perurans* cells detected (Figure 1c). The significance of the interaction between DO treatment and time since inoculation shows that the influence of DO treatment differs with time since inoculation (Figure 1).

### 2.2. Mortality

Daily mortality rates ranged from 0% to a maximum of 26% in the normoxic treatment and 0% to 30% in the hypoxic treatment. Through day three, mortality was very low in both treatments. On day four, cumulative mortality was 5 ± 2% in the hypoxic treatment and 1 ± 1% in normoxic. By the end of day seven, 16 ± 4% of fish in the hypoxic treatment had died compared to 8 ± 2% in the normoxic treatment. On day eight there was a large mortality event in both treatments, with 23% of the remaining fish in the hypoxic treatment dying, and 17% of the normoxic treatment. On day nine mortalities remained high in the normoxic treatment but decreased in fish exposed to hypoxia. At termination of the trial, 10 days post-inoculation, cumulative mortalities were 60 ± 2% in the hypoxic treatment and 53 ± 11% in the normoxic treatment.

### 2.3. AGD Progression

**Figure 2:**
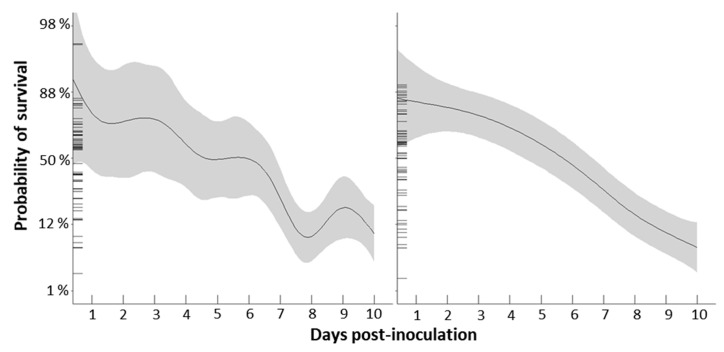
Influence of DO treatment and time since inoculation on probability of survival. Solid lines and shaded areas display the fitted GLMM with 95% confidence intervals.

Probability of survival was significantly influenced by DO treatment and time since inoculation with *N. perurans* (Table 2). Together, the included variables explained 76.8% of deviance observed in the data. Overall, probability of survival decreased with increasing time since amoeba inoculation, but the nature of the decrease differed with DO treatment as evidenced by the significant DO treatment × time since inoculation interaction. In the normoxic DO treatment, probability of survival was relatively stable until five days post-inoculation, after which it decreased consistently until the experiment was terminated (Figure 2). In the hypoxic DO treatment probability of survival decreased in an episodic manner with a large drop between six and eight days post-inoculation (Figure 2). Ultimately, by 10 days post-inoculation, probability of survival was similarly low in both DO treatments.

### 2.4. Gross Lesion Morphometry

There were no differences in mean visible gill surface area, number of lesions, or lesion size between treatments at the termination of the trial. Percentage of visible gill surface area lesioned was 9.0 ± 4.0% in the hypoxic treatment and 6.4 ± 3.7% in normoxic.

### 2.5. Histology

**Figure 3:**
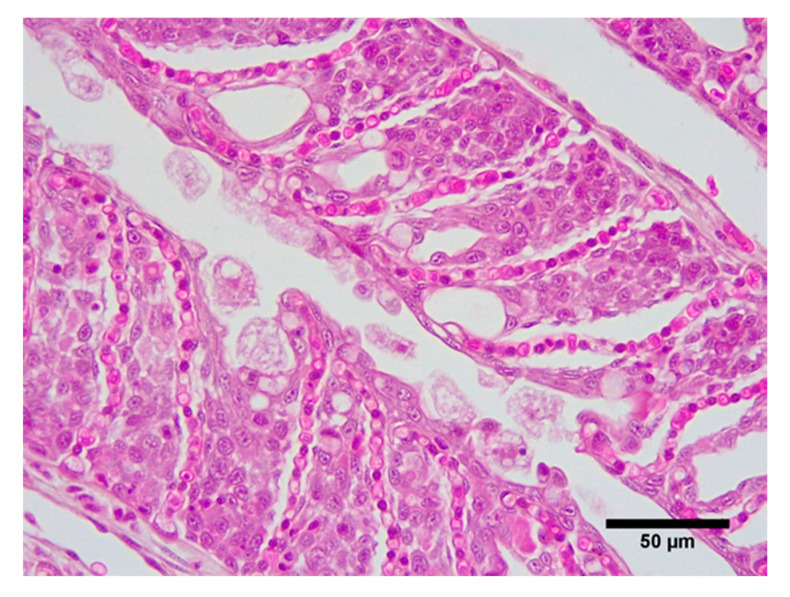
Amoebic gill disease lesion. Extensive epithelial hyperplasia with numerous associated *Neoparamoeba perurans*.

Histological observation confirmed the presence of AGD with severe lesions in both treatments at 10 days post-inoculation, including lamellar fusion and the presence of amoebae (Figure 3). All fish had AGD lesions, and the percentage of filaments with lesions ranged from 25% to 100%. There were no differences in the percentage of filaments with AGD lesions (control = 74 ± 2.8%, hypoxic = 69 ± 3.3%), nor in number of lamellae per lesion (control = 30 ± 0.9%, hypoxic = 27.9 ± 0.9%) at the termination of the trial. The number of amoebae were very high in most cases and AGD lesions extensive. In some cases, lamellar fusion led to the fusion of filaments. An inflammatory response and increase in the number of mucous cells could also be seen in some individuals. Intra-lamellar vesicles were present in many lesions.

## 3. Discussion.

There are three key areas in which hypoxia exposure may alter the impact of AGD on salmon; amoeba acquisition, disease progression, and prognosis. The results of this study show that cyclical exposure to moderate hypoxia did not intensify amoeba acquisition nor lead to poorer prognosis, however it did accelerate disease progression leading to earlier mortalities.

### 3.1. Effects of Hypoxia on Amoeba Acquisition

For salmon, O_2_ uptake relies on the flow of water over the gills. In hypoxic conditions, because there is less DO per volume ventilated, ventilation must increase to obtain the same amount of O_2_. As a result, the volume of water physically contacting gills also increases. In rainbow trout (*Oncorhynchus mykiss*) exposed to gradually decreasing DO over a 6 h period, ventilation rate continuously increased from 70 to 90 breaths min^−^^1^ until DO stabilized [25]. Similarly, mature sockeye salmon (*Oncorhynchus nerka*) held in moderate hypoxia had significantly faster ventilation rates than fish held in normoxic conditions (103 ± 14 min^−^^1^ versus 69.8 ± 6.9 min^−^^1^, respectively) [26]. In salmon, though not quantified, researchers have observed markedly increased gill ventilation in post-smolts with increasing hypoxia severity [27]. Given the opportunistic nature of *N. perurans* and their presence in the water column, it is logical to expect that reduced DO would result in more frequent contact between amoebae and their host, and thus faster disease acquisition [28]. Here, however, this hypothesis did not hold true. Though amoeba numbers were low in both DO treatments 48 h after inoculation, amoeba counts were higher in the normoxic DO treatment than in hypoxic (Figure 1a).

One possible explanation is that fish were exposed to such a high inoculation dose that the impact of hypoxia pre-exposure on acquisition was masked. Time to AGD onset after inoculation, and eventual infection severity, are highly variable and differ with several factors, including *N. perurans* batch, challenge method, stocking density, temperature and inoculation dose [29]. Relative to previous work, we used a moderate inoculation dose and exposure time of 1200 cells L^−^^1^ and 6 h exposure before filtration was restored [29]. Other trials have used up to 9000 cells L^−^^1^ during short-term exposure which resulted in less severe infections, while others have used as few as 300 cells L^−^^1^ and observed AGD related gill pathology at seven days post-inoculation [24,30]. In one of the most comparable *N. perurans* infection studies, amoeba attachment was observed after just 12 h on the gills of salmon which were inoculated with 2600 cells L^−^^1^ for 6 h prior to transfer to new systems. So, while the inoculation dose and challenge method used here were not extraordinary, it is also possible that the amoebae used in this trial were particularly pernicious. Here, amoebae were collected directly from a fish in an AGD maintenance tank and immediately added to the experimental systems, with no intermediate propagation. Given that virulence varies with amoeba batch and decreases with time in culture, it is possible that the amoebae used here were more virulent than those used in previous trials [29,30]. Neither of these explanations, however, explain why amoeba estimates were significantly higher in the normoxic treatment than hypoxic 48 h after inoculation.

A less intuitive but intriguing hypothesis is that the stress response induced by hypoxia had an initially protective effect against *N. perurans* infection. The most effective treatment used commercially for AGD is freshwater bathing, which reduces mucus viscosity in seawater acclimated fish, and is thought to work by removing amoebae from the gill surface [31]. In salmon, up-regulation of mucin transcription in gills has been observed in response to stress [32]. Although no studies have specifically examined mucin production in response to hypoxia in salmon, one experiment found that cortisol, a hormone released during stress, was present at significantly higher concentrations in the plasma of salmon exposed to 50% DO saturation than in normoxic controls [15]. In other fish species, increased mucus production has been observed in response to hypoxia [33], and infestation of rainbow trout (*Oncorhynchus mykiss*) by the parasite *Argulus japonicus* was reduced in fish given an acute dietary cortisol treatment [34]. In this study, perhaps during disease acquisition the stress response initiated by hypoxia increased mucus production and provided an early barrier to infection. Further work, focusing on the inter-relationships between hypoxia, mucus production, and disease acquisition is required to test this hypothesis.

In the context of this study, the higher amoeba numbers initially observed in the normoxic treatment confirm that the difference observed later in the trial was not the result of faster acquisition of amoebae by the salmon in the hypoxic treatment.

### 3.2. Effects of Hypoxia on Disease Progression and Prognosis

AGD is a proliferative gill disease, and though it begins with small lesions it can rapidly escalate, as shown here. Untreated, AGD results in filament fusion and accumulation of mucus on gills, reducing O_2_ uptake capacity and eventually resulting in death [22,35]. In this trial, the trajectory of disease progression differed significantly with DO treatment. Despite equal inoculation pressure and faster acquisition in the normoxic treatment, by 7 days post-infection both the number of amoebae present on gills and cumulative mortality were nearly two times higher in the hypoxic treatment than normoxic.

These results align with those of previous work which found that when exposed to moderate hypoxia, 79% of salmon severely affected by AGD infection died while only 11% of fish with low severity of AGD lesions succumbed [36]. As AGD severity increases, functional gill surface area is reduced, and thus so too is the aerobic scope for activity and capacity to cope with challenging conditions [22]. Another study observed 30% mortality in AGD affected fish after five minutes of confinement, compared to 0% in unconfined fish [37]. Even more striking, mortality increased to 85% when the fish were forced to swim at 1.5 BL s^−^^1^ during recovery. As such, the correlation observed herein between more rapidly developing AGD severity in the hypoxic treatment and earlier mortality is unsurprising.

Less straightforward is why, despite harboring fewer amoebae two days post-inoculation, the hypoxic group would have twice the amoebae of the normoxic group five days later. Hypoxia exposure activates the hypothalamic–pituitary–adrenal (HPA) axis in fish and the subsequent release of cortisol leads to down-regulation of the immune system, which can be problematic when facing chronic stress [12,15,38]. Energetic resources are continuously redirected to cope with a stressor, leaving less available for the immune system to utilize in response to pathogen exposure [39]. In channel catfish (*Ictalurus punctatus*) exposed to two hours of hypoxia and then challenged with *Edwardsiella ictaluri*, plasma bactericidal activity was significantly lower in the hypoxia group compared to normoxic controls [17]. Similarly, in sea bass, *Dicentrarchus labrax*, specific antibody response was significantly reduced after hypoxia exposure compared to fish held in hyperoxia or normoxia [40]. The consequences of such immunocompromise can be severe. For the channel catfish, cumulative mortality in the hypoxic group at 14 days after challenge was more than three times higher than in the normoxic group [17]. Similarly, when yellowtail (*Seriola quinqueradiata*) were challenged with *Enterococcus seriolicida*, mortalities began six days earlier in fish pre-exposed to hypoxia and reached 15% before the first fish held in normoxia died, regardless of DO conditions after pathogen exposure [41].

In this trial, despite the accelerated disease progression in the hypoxic treatment, there was no difference in cumulative mortality at termination 10 days post-inoculation, when more than half of the fish had died in both the hypoxic and normoxic treatments (Figure 2). These findings are similar to those of another study in Atlantic salmon which observed that disease onset and mortality due to infectious pancreatic necrosis virus (IPNV) infection occurred earlier in fish with cortisol implants than those without, but that there was no difference in cumulative mortality at the conclusion of the trial [18]. One possible explanation for this phenomenon is that while chronic stress, induced either artificially or by hypoxia exposure, may reduce the energetic resources available for immune function leading to faster disease progression, in the end the high virulence of the pathogens eventually also cause the un-stressed fish to succumb. Mortalities greater than 80% from untreated AGD have been reported [42], and while prevention measures have nearly eradicated IPNV in farmed salmon, if acquired the disease is often fatal and results in high mortality (70%) [43]. Thus, in this trial, perhaps while AGD impervious individuals survived irrespective of treatment, susceptible individuals succumbed sooner as a result of hypoxia exposure.

### 3.3. Industry Implications

Sub-optimal DO conditions have been documented in marine aquaculture cages around the world, ranging from fluctuating and moderate [2,3,7,44] to constant and extreme [4]. These results demonstrate that exposure to hypoxic conditions can lead to faster disease progression and earlier mortality. In this study, just one week after exposure to *N. perurans,* 16% of fish in the hypoxic treatment had died. However, unlike in this controlled experiment, in marine cages *N. perurans* and DO fluctuations are not the only stressors present. Fish in marine cages face a constantly fluctuating environment where numerous chemical, physical, and biological factors vary simultaneously. During summer and autumn, when AGD and hypoxia risks are greatest, temperatures are also highest, plankton blooms are common, and biofouling rapidly accumulates on cages [45,46,47,48]. Such additional stressors would likely lead to even worse outcomes than observed in this trial. Thus, when AGD and hypoxia co-occur, faster more aggressive treatment is required to minimize mortalities, and during high-risk periods, early detection of disease and knowledge of site-specific DO fluctuations are critical.

To accurately gauge the most extreme conditions experienced by the fish, DO should be monitored inside cages at night or during the early morning, when DO levels are lowest due to lack of photosynthetic activity [49]. During high-risk periods for pathogens, such as summer and autumn for AGD and complex gill disease (CGD), gill checks should be performed frequently to facilitate early detection [21,50,51]. In the event of an infection, stress and handling of fish should be minimized, and efforts made to optimize DO conditions such as removing any cage modifications which reduce water flow [52,53,54], or use of aeration or oxygenation devices.

## 4. Materials and Methods

### 4.1. Animals & Husbandry

Hatchery-reared Atlantic salmon post-smolts were maintained in six independent 650 L recirculating systems with biofiltration, foam fractionation, and ultraviolet (UV) disinfection at the University of Tasmania’s aquaculture research facility (Launceston, Australia). Each system consisted of a 300 L holding tank and 350 L sump. Throughout the trial, all fish were maintained on a 12:12 light/dark cycle at 18 °C and 35 ppt salinity, and fed in excess twice daily at 08:30 and 18:30. Temperature was set at 18 °C to mimic summer temperatures frequently observed in salmon cages, when AGD and hypoxia are most common. Temperature, DO saturation, and salinity were checked daily in each system using an OxyGuard Handy Polaris 2 (OxyGuard A/S, Farum, Denmark) and refractometer, respectively. Nitrate, nitrite, total ammonia, and pH were regularly tested and maintained at NO_3_ ≤ 160, NO_2_ ≤ 5.0, NH_3_ ≤ 3.0 mg L^−^^1^, and pH = 8.1. At the beginning of the trial, each tank contained 32–33 individuals. Throughout the acclimation period from 19–27 July 2017, DO in all systems was ≥90% saturation. Approval for the ethical use of animals in this study was obtained from the University of Tasmania Animal Ethics Committee, permit A0015207.

### 4.2. Hypoxia Induction

**Figure 4:**
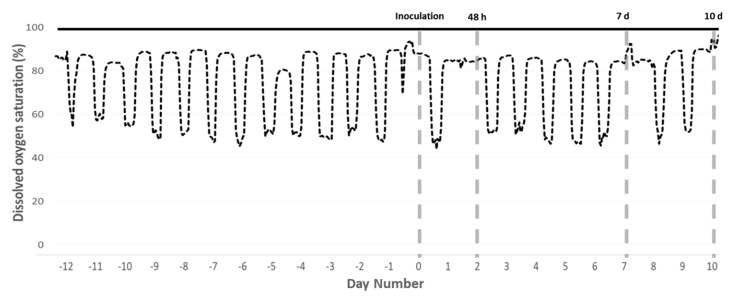
Experimental timeline. Diel cycling hypoxia began in the hypoxic treatment tanks (dashed black line) 12 days before inoculation with *N. perurans*. Normoxic control tanks (solid black line) were maintained above 90% DO saturation throughout. Timing of inoculation and the three sampling events is indicated by grey dashed lines. Data presented are from representative tanks.

On 28 July 2017, the cyclic hypoxia regime commenced (Figure 4), with triplicate systems for each of the two treatments assigned in a randomized block design. A control group of three systems was maintained at ≥90% DO saturation (normoxic treatment), and three systems were subjected to cyclic hypoxia (hypoxic treatment).

Cyclic hypoxia was chosen to mimic conditions in marine cages, where DO typically fluctuates daily due to changes in photosynthetic O_2_ production [3,49]. Daily at 09:00 the pumps were turned off and circulation discontinued in all six systems. In the three normoxic tanks, aeration supplied through a ceramic bubble generation system was set to the minimal flow rate required to maintain DO ≥ 90% saturation. In the three hypoxic treatment tanks, aeration was also discontinued and DO allowed to drop until 50% saturation was reached, at which point aeration was restored at the appropriate flow rate to maintain hypoxic conditions [15]. Because aeration was manually controlled, DO during hypoxic periods fluctuated, but was held between 40% and 60% saturation. DO measurements in marine cages are highly variable, both spatially and temporally, but multiple studies have documented salmon exposure to DO of less than 50% saturation for several hours [3,4,55]. At 18:30 all pumps were turned back on and circulation restored, returning all systems to ≥90% DO saturation.

### 4.3. Amoeba Inoculation

Gills removed post-mortem from a salmon held in an AGD infection tank were used to isolate *N. perurans* (University of Tasmania, Launceston, Australia) as previously described [56]. Amoebae were enumerated and tested for viability following the procedure utilized by [29].

On 9 August 2017 (day 0) all amoebae were pooled and divided evenly into six flasks containing 400 mL of filtered seawater. At 11:00 circulation was discontinued, the water volume in all six systems reduced to 80 L, and amoebae were simultaneously added to all systems resulting in an inoculation dose of 1200 cells L^−^^1^. Throughout the inoculation period, aeration was at maximal flow in all systems and DO saturation was ≥90%. At 17:00 the pumps were turned on, all tanks returned to 300 L volume and circulation restored.

### 4.4. Sampling Protocol

Fish were not fed for 24 h prior to sampling. On day 2, 48 h after inoculation with *N. perurans*, the first non-lethal sampling was performed. Twelve live fish were transferred by hand-net from their tank into a 100 L bin with light anesthetic (20 mg L^−^^1^ clove oil) and aeration. After 10 min, two fish at a time were transferred into a bin with full strength anesthetic (40 mg L^−^^1^ clove oil) and aeration. When fish became unresponsive to touch, they were removed from the bin and a single swab, with a 1/4 rotation between each arch, was swept across the front of all four arches in the left gill basket. Swabs were immediately placed in 500 µL lysis solution (7.8 M Urea, 0.5% SDS, 10 mM Tris and pH 7.5). After sampling, each fish was marked on its dorsal side just above the anal fin with a sub-dermal injection of Alcian blue dye and returned to its tank. Normoxic systems were sampled first, the water changed in both anesthetic baths, and then hypoxic systems were sampled.

On day 7 a second non-lethal sampling was performed, and the left gill arches of all surviving unmarked fish were swabbed using the previously described procedure. Hypoxic systems were sampled first, the water changed in both anesthetic baths, and then normoxic systems were sampled.

The final sampling was performed 10 days post-infection (Figure 4). All surviving fish were pre-anesthetized with 20 mg L^−^^1^ clove oil before being transferred to a bin with full strength anesthetic of 40 mg L^−^^1^ clove oil. For each individual, fork length and weight were measured, and skin, fin, and eye condition visually scored. Physical condition was qualitatively scored from 0 (no abnormality) to 3 (severe). Fish were then killed by cervical transection and the right gill basket was removed. The 2nd right arch was stored in 750 mL RNA stabilization reagent (25 mM sodium citrate, 10 mM EDTA, 10 M ammonium sulphate and pH 5.2), and the 3rd and 4th right arches were placed in seawater Davidsons fixative for 36 h before transfer to 70% ethanol. For fish that died outside sampling events, visual observations were performed to inspect gills and physical condition, but because time of death was unknown no samples were collected.

### 4.5. Gross Lesion Morphometry

Following fixation, macroscopic images were taken of the anterior surface of the 3rd right arch and used to assess lesion number and affected surface area. Gill arches were photographed submerged in a 70% ethanol solution against a white grid background with fixed lighting. Using the ImageJ software program (U.S. National Institutes of Health, Bethesda, MD, USA), total visible gill surface area and surface area of all lesions (affected area) were measured [57]. This measure provided an approximation of the proportion of total gill affected.

### 4.6. Histological Analysis

After imaging, gill arches were dehydrated in a graded ethanol series, embedded in paraffin, sectioned (5 µm) and stained with hematoxylin and eosin (H&E). The sections were examined at 100× and 400× magnifications under a bright field light microscope (Leica DM1000, Hamburg, Germany). Percentage of filaments affected by AGD lesions and number of lamellae in each lesion were determined from the sections. The number of lamellae in lesions was determined from eight lesions from each fish. Lesions were chosen using random selection procedures. Only filaments cut at a correct angle (with equal length of lamellae with typical morphology) were included in the analysis. These measures confirmed presence of *N. perurans* and AGD, allowing a direct comparison of severity between this study and previous work.

### 4.7. N. Perurans Enumeration

Twenty-four hours before DNA extraction, arches were transferred from the RNA stabilization reagent into 1 mL lysis solution and stored at 4 °C. DNA was extracted from all samples (arches and swabs) using the same protocol. After vortexing, 250 µL of sample solution was combined with 250 µL lysis solution and 2 µL proteinase K (20 mg mL^−^^1^). The mixture was then incubated at 37 °C for 30 min. After incubation, samples were chilled on ice for 5 min, combined with 250 L 7.5 M ammonium acetate, and vortexed for 20 s. Samples were then centrifuged at 14,000× *g* for 5 min and the resulting supernatant combined with 750 µL isopropanol. To facilitate precipitation, samples were then inverted for 5 min and centrifuged at 16,000× *g* for 10 min. The nucleic acid pellets were rinsed twice in 70% ethanol and re-suspended in 100 µL buffer (10 mM Tris, 0.05% Triton ×100).

A real-time PCR assay which distinguishes an *N. perurans*-specific 18S rRNA gene sequence [58] was run on all samples, in duplicate, with no-template controls. All reactions were run in a CFX Connect PCR Detection System (Bio-Rad, Hercules, CA, USA). *N. perurans* cell numbers were estimated based on 2880 copies cell^−^^1^ as previously determined [58]. This method allowed non-lethal sampling throughout disease progression, and quantitative comparison of infection severity between treatments.

### 4.8. Data Analyses

To evaluate the impact of DO treatment on amoeba acquisition and proliferation, a negative binomial generalized linear mixed model (GLMM) was created with the package ‘glmmTMB’ [59] in R [60]. The number of *N. perurans* cells detected via qPCR was modelled as a function of DO treatment (hypoxic or normoxic) and time since inoculation (48 h, 7 d, 10 d), as well as their interaction, DO treatment × time since inoculation. To incorporate the dependency among observations of the same tank, we applied a mixed model with tank as random intercept. A Poisson distribution, as is typically used for count data, was tested, however due to over-dispersion the negative binomial distribution was a more appropriate fit for these data. Model assumptions were verified by plotting Pearson residuals versus fitted values and versus each covariate.

To examine the impact of DO treatment on AGD progression, a binomial generalized additive mixed model (GAMM) was created using the package ‘mgcv’ [61] in R. Probability of survival was modelled as a function of DO treatment (hypoxic or normoxic) and days post inoculation (continuous), as well as their interaction, DO treatment × days post inoculation. A cubic regression spline smoother was applied to the DO treatment × days post inoculation interaction. To incorporate the dependency among observations of the same tank, tank was included as a random effect. Model assumptions were verified by plotting Pearson residuals versus fitted values and versus each covariate. Data presented are mean ± standard error unless otherwise stated.

## 5. Conclusions

Despite the widespread and predictable occurrence of hypoxia in marine cages, and the numerous pathogens which threaten farmed salmon, little attention has been given to the impact of hypoxia exposure on the prevalence and intensity of infectious diseases. This study provides the first evidence that exposure to hypoxia accelerates disease progression in Atlantic salmon, even when fish are exposed to only moderately hypoxic conditions for a few hours daily. More extreme consequences could be expected as a result of more severe hypoxia exposure or the presence of other stressors.

## Figures and Tables

**Table 1:** Estimated regression parameters, standard errors, *z*-values and *p*-values for the negative binomial GLMM of amoeba number as a function of DO treatment and time since inoculation.

	Estimate	Std. Error	*z*-Value	*p*-Value
Intercept	0.920	0.194	4.736	<0.001
Treatment_Normoxic	0.718	0.264	2.721	<0.01
7 days post-inoculation	7.232	0.240	30.102	<0.001
10 days post-inoculation	6.361	0.238	26.659	<0.001
Treatment_Normoxic: 7 days	−1.258	0.328	−3.826	<0.001
Treatment_Normoxic: 10 days	−1.133	0.325	−3.482	<0.001

**Table 2:** Estimated regression parameters, effective degrees of freedom, chi squared and *p*-values for thebinomial generalized additive mixed model (GAMM) of amoeba counts as a function of sample period and DO treatment.

	Estimate	edf	Chi.sq	*p*-Value
Intercept	3.7609			
Day: Hypoxic		6.478	54.666	<0.001
Day: Normoxic		2.593	57.605	<0.001
Tank		2.074	3.858	0.103
